# 1166. Reverse Syphilis Screening and Adherence to The Congenital Syphilis Guidelines: Institutional Experience

**DOI:** 10.1093/ofid/ofab466.1359

**Published:** 2021-12-04

**Authors:** Brandon Chatani, Aida Chaparro, Patricia Alvarez, Kristopher Arheart, Ivan Gonzalez, Gabriela Moraru

**Affiliations:** 1 University of Miami/Jackson Health System, Miami, Florida; 2 University of Miami, Miami, Florida; 3 Jackson Health System /Holtz Children’s Hospital, Miami, Florida

## Abstract

**Background:**

This study is analysis the consequences of the reverse syphilis screening on the management of newborns exposed to maternal syphilis, and pediatric physicians’ adherence to the existing guidelines.

**Methods:**

We conducted a 5-year retrospective review of the maternal population and their newborns diagnosed with syphilis. Women with positive results (TT+/NTT+) and discordant (TT+/NTT-/TT+) and their newborns were included in the analysis.

**Results:**

Per American Academy of Pediatrics (AAP), the 202 newborns were divided in two groups: proved or highly probable and possible congenital syphilis (Group A, n=102) and less likely and unlikely congenital syphilis (Group B, n=100). Except for the RPR, none of the other laboratory tests showed higher odds for predicting congenital syphilis. The RPR titers above 1:16 were only identified among newborns belonging to the Group A (5%); 32 patients (31%) in the Group A and 19 (9%) in the Group B had an RPR titer equal to or below 1:8. An RPR titer equal to or above 1:4 was almost three times more likely to be identified in patients from Group A (OR 2.91; CI 1.51- 5.59, p< 0.05). The newborns with non-reactive RPRs represented 64% of the patients in the Group A and 47% of them were born to mother with non-reactive RPR also (mothers with discordant results). Among the Group B, 82% of the neonates had a non-reactive RPR and 54% were delivered to mother with non-reactive RPRs. Babies in Group B had additional work-up performed 69% (n=37) of the time; 15% of these babies were treated with intramuscular penicillin which does not follow established AAP guidelines.

Statistical analysis of the laboratory tests used for the congenital syphilis work-up

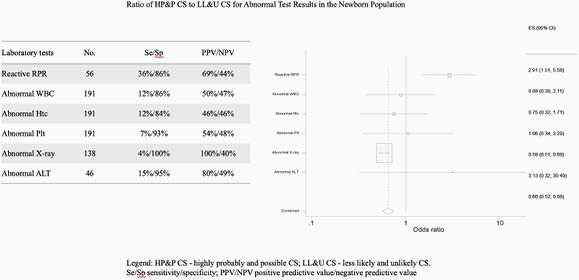

Result table comparing the two groups of newborns

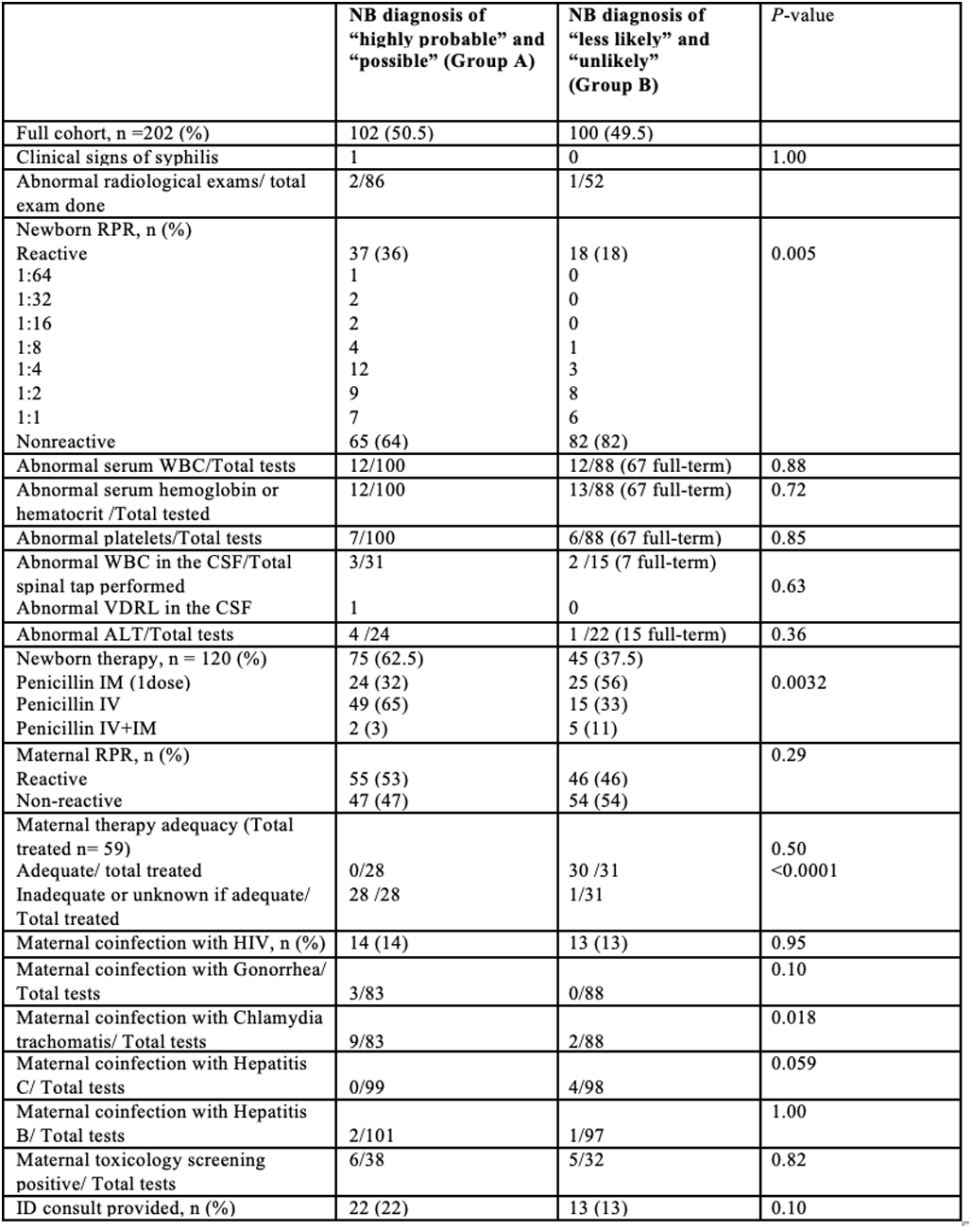

**Conclusion:**

The reverse syphilis screening and non-adherence to the guidelines led to additional screening to half of the newborns in both groups. This study highlights the need for a comprehensive maternal history at the time of delivery that is effectively communicated between the providers. This might lead to greater congruence with the established AAP guidelines.

**Disclosures:**

**All Authors**: No reported disclosures

